# A Comprehensive Analysis of Metabolomics and Transcriptomics in Cervical Cancer

**DOI:** 10.1038/srep43353

**Published:** 2017-02-22

**Authors:** Kai Yang, Bairong Xia, Wenjie Wang, Jinlong Cheng, Mingzhu Yin, Hongyu Xie, Junnan Li, Libing Ma, Chunyan Yang, Ang Li, Xin Fan, Harman S. Dhillon, Yan Hou, Ge Lou, Kang Li

**Affiliations:** 1 Department of Epidemiology and Biostatistics, School of Public Health, Harbin Medical University, Harbin, 150086, P.R. China; 2 Department of Gynecology Oncology, the Tumor Hospital, Harbin Medical University, Harbin, 150086, P.R. China; 3 State Key Laboratory of Natural Products, Jiangsu Key Laboratory of TCM Evaluation; Translational Research Department of Complex Prescription of TCM, Pharmaceutical University, 639 Longmian Road, Nanjing 211198, P.R. China; 4 School of Basic Medical Sciences, Heilongjiang University of Chinese Medicine, Harbin, Heilongjiang 150040, P.R. China; 5 Harbin Medical University, Harbin, 150086, P.R. China; 6 Key Laboratory of Cardiovascular Medicine Research, Harbin Medical University, Ministry of Education, Harbin, 150086, P.R. China

## Abstract

Cervical cancer (CC) still remains a common and deadly malignancy among females in developing countries. More accurate and reliable diagnostic methods/biomarkers should be discovered. In this study, we performed a comprehensive analysis of metabolomics (285 samples) and transcriptomics (52 samples) on the potential diagnostic implication and metabolic characteristic description in cervical cancer. Sixty-two metabolites were different between CC and normal controls (NOR), in which 5 metabolites (bilirubin, LysoPC(17:0), n-oleoyl threonine, 12-hydroxydodecanoic acid and tetracosahexaenoic acid) were selected as candidate biomarkers for CC. The AUC value, sensitivity (SE), and specificity (SP) of these 5 biomarkers were 0.99, 0.98 and 0.99, respectively. We further analysed the genes in 7 significantly enriched pathways, of which 117 genes, that were expressed differentially, were mainly involved in catalytic activity. Finally, a fully connected network of metabolites and genes in these pathways was built, which can increase the credibility of our selected metabolites. In conclusion, our biomarkers from metabolomics could set a path for CC diagnosis and screening. Our results also showed that variables of both transcriptomics and metabolomics were associated with CC.

Cervical cancer (CC) is one of the most common types of gynecological malignancies worldwide that is particularly prevalent in the developing countries, with an estimated 485,000 new cases and 236,000 deaths in 2013^1^. Advances in research continue to improve the precautionary methods available in developed countries, therefore, incidence rate vary markedly around the world^2^. In the developed countries, the incidence has decreased due to regular Pap tests and vaccination, which could detect cervical pre-cancer before it progressed into cancer. In the U.S., approximately 12,990 women were diagnosed with cervical cancer and roughly 4,120 women died from it in 2016^3^. However, in China, younger women showed an increasing trend during the period of 1988–2002, especially in women residing in rural areas, although, the incidence and mortality rates declined during the same period in elder women^4^. As we know, screening and early diagnosis of cervical cancer is crucial for the prognosis of patients. The most widely known biomarker for CC is squamous cell carcinoma antigen (SCC-Ag), which is a tumor-associated antigen identified by Kato *et al*. in 1977^5^. SCC-Ag was elevated in 50% of patients with stage I disease, 71% with stage II and 82% with stage III-IV^6^. From these results, we can see that the positive detection rate is low in early stages. Although, circulating antibodies and mRNA have been investigated in the potential biomarkers for CC^7^,^8^, the diagnostic accuracy and predictive performance are still under debate.

Metabolomics have been widely used in cancer metabolism and biomarker identification to infer the onset and progression of cancer^9^. Metabolites, the final products of various biological processes, hold promise as accurate biomarkers that reflect upstream biological events such as genetic mutations and environmental changes^10^. Altered metabolites and pathways would help better understand dysregulated metabolism in tumor initiation and progression^11^. Some metabolomics studies have been applied to CC^12^,^13^,^14^,^15^. For examples, Hasim *et al*. reported a profiling of CC for 19 amino acids^16^ and Yin *et al*. identified 4 lipids as new biomarkers for CC^17^. But the sample sizes of these studies were relatively small, which would decrease the credibility of the study and limit the clinical application of biomarkers.

Similar to other types of biomarkers, metabolomic biomarkers are difficult to replicate across different studies. The possible reasons mainly attribute to the population heterogeneity and sample sources, different experimental protocols, parameters setting in the metabolomics data, as well as biological variations in the turnover rates of metabolites^11^. All of these limitations have resulted in little progress in introducting new cancer biomarkers into clinical practice. Due to the development of system biology and bioinformatics tools, integration of metabolomic profiling with transcriptomics data (expression profiling by array) has been recently used in cancer research and may yeild further insight into these fields than either approach alone^18^. This new approach could investigate pathogenesis from a view of system biology and improve the credibility of biomarkers. To date, no study has aimed at exploring cervical cancer deeply through integration of metabolomics and transcriptomics with large samples.

So, in order to investigate the dysregulated pathways and identify more reliable biomarkers for cervical cancer, we performed a comprehensive analysis of metabolomics and transcriptomics. We hypothesized that metabolites and genes that were involved in the same biological processes were often dysregulated together in cancer^11^,^19^. Therefore, integration of metabolomic profiling with transcriptomics data could be used in validating the potential diagnostic biomarkers. Pathway and network analyses were then used to further explore the relationship between our selected metabolites and genes, thus, increasing reliability for our results.

## Results

### Demographic and clinical characteristics

The detailed demographic and clinical characteristics were listed in Table 1. The metabolomics data were separated into training and test sets according to the enrollment time. The training set included 70 CC and 80 NOR cases, and the test set consisted of 66 CC and 69 NOR cases. In total, 47 CC patients were classified as stage I, 64 as stage II, and 1 as stage III. The SCC-Ag levels of 53 CC patients were in the reference range (0–1.5), and 78 were above the reference range. The transcriptomics data composed of 28 CC and 24 NOR cases.

### Metabolic profiling of CC and NOR

In this study, non-targeted LC-MS-based metabolomics detection was used. After deducting the isotope peaks, 3495 ions in the ESI+ mode and 3052 ions in ESI- mode were detected. Two-dimensional PCA score plots of all samples, in both the ESI+ and ESI- modes, revealed no outliers in this study, and the tightly clustered QC samples ensured detection stability (see Supplementary Fig. S1).

**Figure 1:**
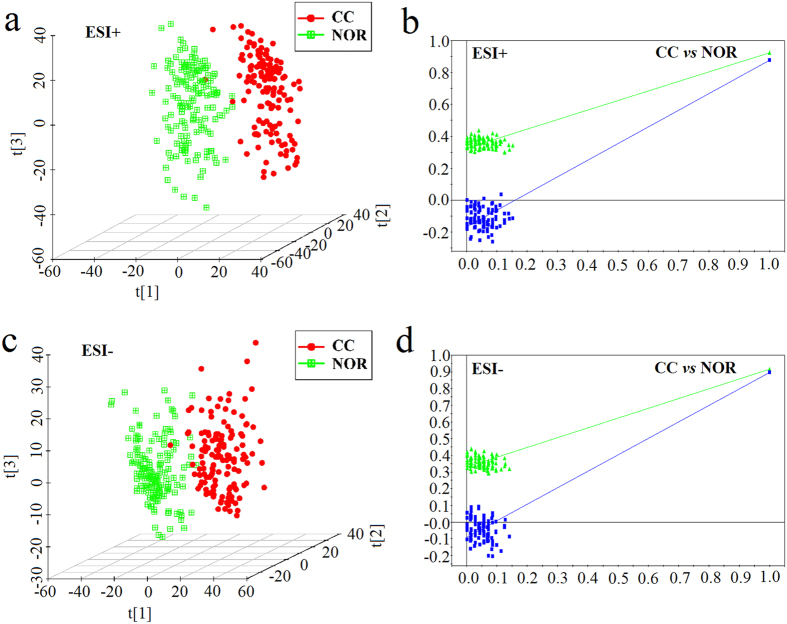
PLS-DA three-dimensional score plots and validation plots for the metabolic profiling results. (**a**) PLS-DA three-dimensional score plot for CC *versus* NOR in the ESI+ mode (three latent variables, R2X = 0.211, R2Y = 0.924, Q2 = 0.878). (**b**) Validation plot for CC *versus* NOR in ESI+ mode. (**c**) PLS-DA three-dimensional score plot for CC *versus* NOR in the ESI- mode (three latent variables, R2X = 0.297, R2Y = 0.917, Q2 = 0.896). (**d**) Validation plot for CC *versus* NOR in ESI- mode. The criteria for stability and credibility are as follows: all permuted R2 and Q2 values on the left are lower than the original point on the right, and the Q2 regression line in blue has a negative intercept.

Three-dimensional PLS-DA score plots revealed a significant difference in metabolism mode for CC and NOR (Fig. 1a and c). The cumulative R2Y and Q2 were 0.924 and 0.878, respectively, for CC and NOR in the ESI+ mode when the first three components were calculated. The two values in the ESI- mode were 0.917 and 0.896. Validation plots obtained from 100 permutation tests showed that our PLS-DA models prevented overfitting and they were stable and credible (Fig. 1b and d). The stability and credibility were supported by the result that all permuted R2 and Q2 values on the left were lower than the original point on the right, and that the Q2 regression line in bule had a negative intercept^20^.

### Differential metabolites between CC and NOR

In total, 34 metabolites in the ESI+ mode and 28 metabolites in the ESI- mode met the standard of lfdr < 0.05 and VIP > 1. The detailed statistical and biological information of these metabolites were listed in Supplementary Tables S1 and S2. Boxplots of all metabolites were presented in Supplementary Fig. S2, within which, 55 metabolites were down-regulated in CC patients while 7 metabolites were up-regulated.

**Figure 2:**
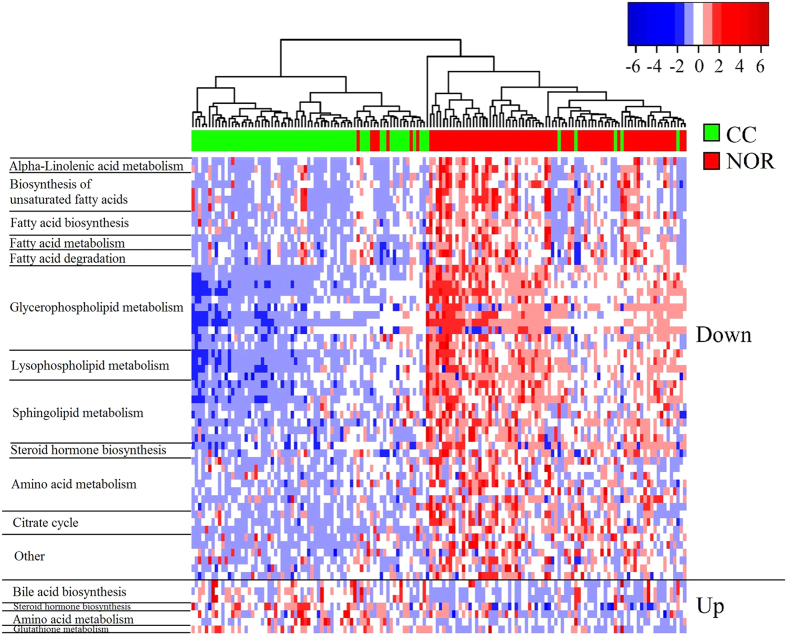
HCA-heatmap plot of 62 differential metabolites between CC and NOR. Down indicated that these metabolites were down-regulated in cervical cancer patients, Up indicated that these metabolites were up-regulated in cervical cancer patients.

The HCA-heatmap for the 62 differential metabolites between CC and NOR were presented in Fig. 2. In the HCA-heatmap diagram, CC were separated from NOR, with the exception of 5 CC that were wrongly clustered with NOR and 7 NOR that were falsely clustered with CC.

### Biomarkers for cervical cancer diagnosis

By clustering metabolites based on their metabolic profiling, we obtained a total of 5 clusters (see Supplementary Table S3). According to the selection principle mentioned in methods section, we selected 5 metabolites as candidate biomarkers for cervical cancer, including bilirubin, LysoPC(17:0), n-oleoyl threonine, 12-hydroxydodecanoic acid, tetracosahexaenoic acid. The AUC value, sensitivity (SE) and specificity (SP) of these biomarkers were 0.99, 0.98, and 0.99, respectively (Table 2).

### Pathway analysis

The 62 differential metabolites between cervical cancer patients and normal controls were used for pathway analysis conducted by MetaboAnalyst 3.0. A total of 31 pathways were enriched, of which 7 pathways were enriched significantly. The seven pathways consisted of the fatty acid biosynthesis, glyoxylate and dicarboxylate metabolism, citrate cycle, lysine biosynthesis, histidine metabolism, lysine degradation, and steroid hormone biosynthesis (see Supplementary Fig. S3 and Supplementary Table S4).

These pathways were mainly involved in carbohydrate metabolism (citrate cycle, glyoxylate and dicarboxylate metabolism), lipid metabolism (fatty acid biosynthesis, steroid hormone biosynthesis), and amino acid metabolism (lysine biosynthesis, histidine metabolism, lysine degradation), which played important roles in the rapid growth of cancer tissue and metastasis of cancer cells. The up-regulated L-thyroxine was involved in tyrosine and significant down-regulation of metabolites related to the citrate cycle and fatty acid metabolism resulted in rapid but inefficient energy metabolism. The rapidly proliferating cells required ATP as well as nucleotides, proteins, fatty acids, and membrane lipids, which could also explain the down-regulation of metabolites involved in these pathways.

### Transcriptomics data analysis

**Figure 3:**
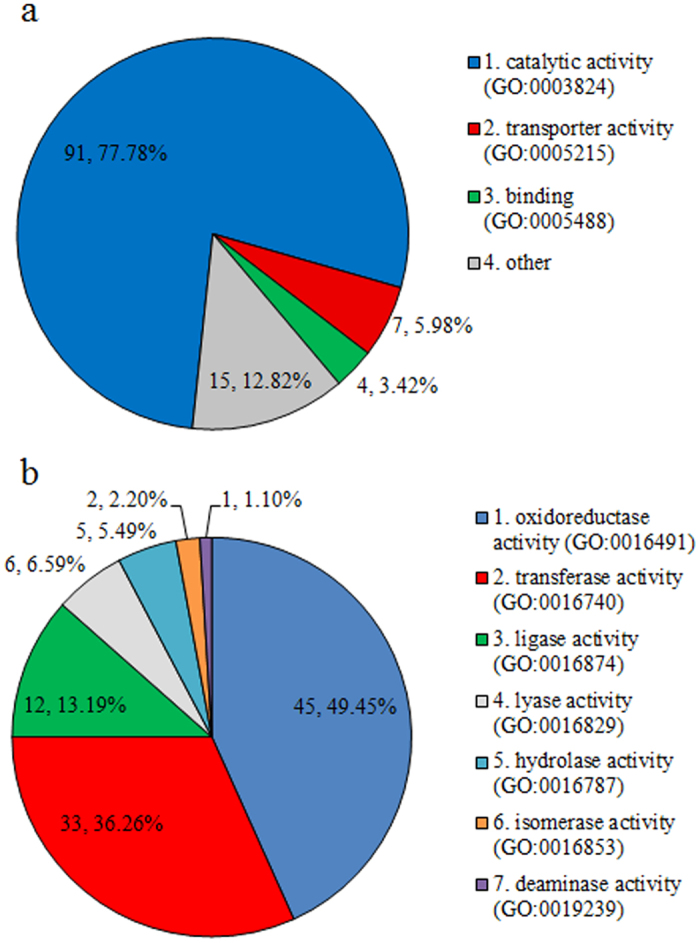
Pie chart of gene functions in 7 pathways. (**a**) Pie chart of PANTHER GO-slim molecular function of 117 genes. (**b**) Pie chart of 91 genes who have the function of catalytic activity (some gene may have more than one function, so the sum of genes is not 91).

We further analyzed genes in 7 pathways with P < 0.1. Among a total of 181 genes in these pathways, 117 genes (64.64%) were differentially expressed with lfdr < 0.05, in which, most genes (91, 77.78%) present with function of catalytic activity (Fig. 3a). We further analysed the molecular function of genes with catalytic activity and found that they were mainly involved in oxidoreductase activity (45, 49.45%), transferase activity (33, 36.26%) and ligase activity (12, 13,19%) (Fig. 3b). The lfdr, function and pathway information of genes were listed in Supplementary Tables S5, S6 and S7.

### Network analysis of differential metabolites and genes

**Figure 4:**
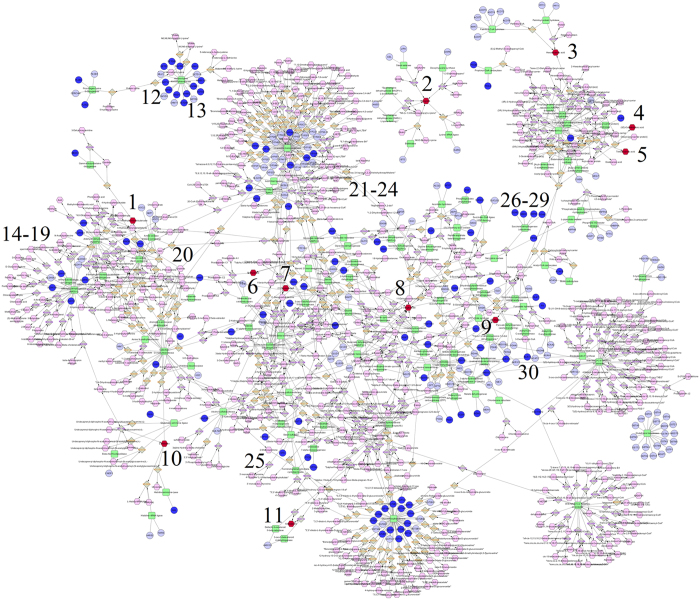
Fully connected network of metabolites and genes in our selected 7 pathways. The nodes in red indicated differential metabolites (1–11) and the nodes in blue indicated differentially expressed genes (12–30) in this study. The nodes in green indicated enzymes in these pathways. 1. 4-Trimethylammoniobutanoic acid. 2. L-Lysine. 3. Palmitic acid. 4. Oleic Acid. 5. Myristic acid. 6. L-Glyceric acid. 7. 21-Deoxycortisol. 8. Oxoglutaric acid. 9. L-Malic acid. 10. L-Histidine. 11. Aldosterone. 12. WHSC1. 13. EHMT2. 14–19. ALDH1B1, ALDH2, ALDH3B1, ALDH3B2, ALDH7A1, ALDH9A1. 20. MAOA. 21–24. CYP1A2, CYP2E1, CYP3A4, CYP19A1. 25. STS. 26–29. SDHA, SDHB, SDHC, SDHD. 30. ACAT1.

Fully connected network of metabolites and genes in our 7 selected pathways were built with Metscape (Fig. 4). Although, the metabolomics and transcriptomics data in our study were generated from different populations and technology platforms, a lot of metabolites and genes in the same pathways were found differentially in the network of these pathways. This result can further increase the credibility of our selected metabolites, genes, and pathways.

## Discussion

In our study, a large population of cervical cancer patients was enrolled to explore the metabolic characteristics and biomarkers of this cancer through the metabolomic strategy. The selected metabolites and corresponding pathways were then validated by transcriptomics data from GEO. Furthermore, five biomarkers were selected as candidate biomarkers for cervical cancer diagnosis, the combination of which resulted in an AUC value of 0.99, an SE of 0.98, and an SP of 0.99 and could be a promising method for cervical cancer diagnosis and screening.

Based on our results, we can easily infer that significant changes, related to energy metabolism, occurred in patients with cervical cancer. The decreased metabolites (L-malic acid, oxoglutaric acid, pyruvate) in citrate cycle supported the hypothesis that ATP generation, through oxidative phosphorylation in the mitochondrion, was shifted to ATP generation through glycolysis in the cytoplasm^21^. ACAT1 and ACSBG1, which encoded enzyme responsible for the catalyzed the reactions of acyl-CoA, were found to be down regulated. Four succinate dehydrogenase (SDHA, SDHB, SDHC, SDHD), which may associated with mitochondrial dysfuncton and tumorigenesis, were also dysregulated. All these findings, from transcriptomics data, could support our metabolomics study. Studies have shown that the oxidative phosphorylation was affected by uncoupling proteins (UCPs), including a mitochondrial inner membrane protein^22^. UCPs can eliminate the proton gradient, slow down oxidative phosphorylation and hinder the production of ATP. UCPs were increased by L-thyroxine, which was increased in CC^23^. Glycolysis had the capacity to generate ATP more rapidly than oxidative phosphorylation, providing energy for rapid cell division of cancer tissues, although, glycolysis was far less efficient than oxidative phosphorylation at generating ATP^21^. These findings may indicate up-regulated gluconeogenesis from lipids and proteins, which were consistent with the down-regulated lipids and amino acids in plasma. All of these results were consistent with the Warburg effect and inefficient energy metabolism in tumor tissues.

A series of glycerophospholipids (LysoPCs and LysoPEs) and sphingolipids (e.g. Cers, CerPs, sphinganine) was also down-regulated in the plasma of cancer patients. All of these molecules were lipids and had many important bio-functions. It is well-known that LysoPCs, which were formed by hydrolysis of membrane phosphatidylcholine (PC), can induce inflammation, apoptosis, and tumor cell invasiveness^24^. Cers were also a kind of signaling molecule related to inflammation and apoptosis^25^,^26^. So the tumor cells may down-regulate the enzymes related to the production of inflammatory lipids (LysoPCs and Cers), preventing damage from the immune system.

L-lysine, an essential amino acid, was decreased in CC. L-lysine deficiency may also result in immunodeficiency, improving the proliferation of cancer cells. Decreased 4-trimethylammoniobutanoic acid in lysine metabolism also resulted in a lack of lysine in CC. γ-CEHC was down-regulated in CC as well. They were converted from tocopherols, which were cancer preventive^27^. Research has demonstrated that bilirubin, a breakdown product of hemecatabolism, was decreased in colon cancer patients^28^. This was consistent with our study that bilirubin was down-regulated in CC. So this metabolite may be related to a variety of cancer types.

One hundred and seventeen genes, involved in the pathways of carbohydrate metabolism, lipid metabolism, and amino acid metabolism, were differentially expressed between CC and NOR. These genes were involved in the pathways above and could support our findings in metabolomic research. Network analysis indicated that these differential metabolites and genes were closely connected and the corresponding pathways have been obviously disturbed. A lot of the differentially expressed genes (DEGs) have a function of catalytic activity, including oxidoreductase activity, transferase activity, ligase activity, and so on. This was another piece of evidence proving the disturbances of these pathways.

The genes in our study still have a variety of other important features. AKR1C2, whose overexpression was a high-risk factor in bladder cancer^29^, was also over-expressed in CC. A series of genes (e.g. CYP1A2, CYP3A4, CYP19A1), related to cancer in Cytochrome P450 Family, were down-regulated, with the exception of CYP2E1, which was up-regulated and may be involved in carcinogenic process of cervical cancer^30^. MAOA suppression could be associated with the development of cancer^31^. EHMT2 dysfunction has been proved to be involved in the autophagy-associated cell death and EHMT2 inhibition can be an effective threpeutic strategy for cancer treatment^32^. Increased sulfatase (STS) activity was associated with a worsening progression in patients with breast and ovarian cancer and it would be a potential therapeutic target in the treatment of cancer^33^,^34^. Aldehyde dehydrogenase (ALDH1B1, ALDH2, ALDH3B1, ALDH3B2, ALDH7A1 and ALDH9A1) were involved in the resistance against cyclophosphamide/carboplatin in cancer chemotherapy^35^. WHSC1 may serve as a new molecular marker to predict the prognosis of ovarian cancer^36^. These DEGs may play important roles in the pathogenesis, therapy, and prognosis in CC, thus, further studies were needed to validate their functions in this disease.

The comprehensive analysis of transcriptomics and metabolomics in our study revealed the significant alterations of 7 pathways in cervical cancer at both the transcriptional and metabolic levels. Metabolites were final products of cellular biological processes, which were affected by genetic and environmental factors. While genes and their encoded proteins play an important role in the metabolic process of metabolites, including catalyzing and providing place for the process. Konwing this, transcriptomics study could further validate the metabolomics studies and comprehensive analysis of these two omics data provided a systems level perspective of dysregulated pathways that could facilitate the development of therapy and biomarkers for cervical cancer.

There were several limitations in our study. One problem was that the metabolomics data of CC and NOR were generated from different populations. But we have tried our best to minimize the sample heterogeneity during sample collection, storage, and preparation. The other was that the metabolomics and transcriptomics data in our study were generated from different populations and technology platforms. However, we believed that the differences of metabolomics and transcriptomics data can make our study more reliable.

In summary, we performed a comprehensive analysis of metabolomics and transcriptomics to explore cervical cancer metabolism characteristics. Then, a combination of 5 biomarkers, which had an excellent performance in distinguishing CC and NOR, was established as a promising method for cervical cancer diagnosis and screening. Finally, we explained the aberrant metabolism of cervical cancer at transcriptional and metabolic levels, explored the roles of key genes in cancer, and demonstrated that the comprehensive analysis of metabolomics and transcriptomics was a promising method to investigate the mechanism of carcinogenesis and discover more reliable biomarkers.

## Methods

**Figure 5:**
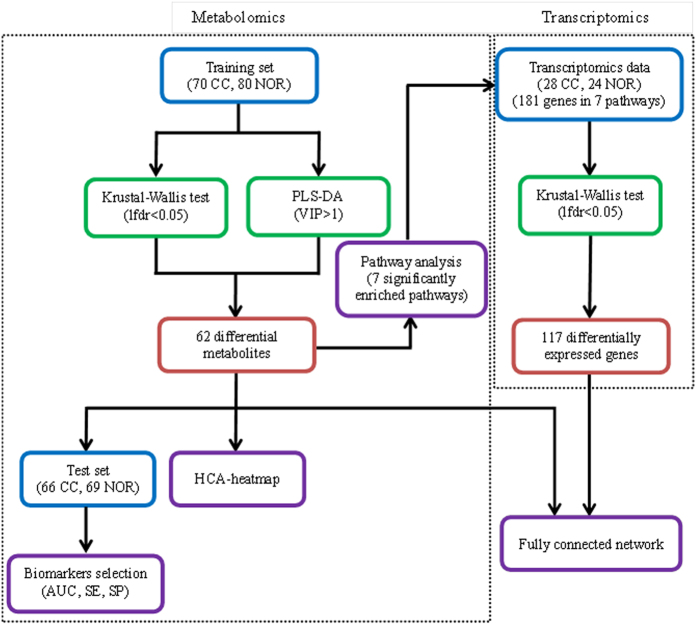
An overview workflow of the comprehensive analysis of metabolomics and transcriptomics in cervical cancer.

The overview workflow of the comprehensive analysis of metabolomics and transcriptomics in cervical cancer was summarized in Fig. 5.

### Study design

This is a prospective study, which collects the plasma from patients suspected of having cervical cancer and from control groups. Plasma samples of cervical cancer patients were collected by the Department of Gynecology of Harbin Medical University Tumor Hospital (Harbin, China). The plasma from control groups were obtained from healthy volunteers from the Daoli district in Harbin, China. The inclusion criterion were as follows: all participants who were pathologically confirmed to have cervical cancer and did not receive any medical intervention for it. The exclusion criterion was as follows: people with metabolic, liver, or kidney diseases, or any other type of cancer were excluded.

### Ethical approval

Our proposal aims to identify the biomarkers related to the early diagnosis, personalized treatment prognosis in cervical cancer and ovarian cancer patients. Our present study is one part of this proposal to identify the potential biomarkers for the early diagnosis of cervical cancer. Informed consents were signed by all participants in this study, which was approved by the ethics committee of Harbin Medical University (Harbin, China). The methods were carried out in accordance with the approved guidelines.

### Data sources

In this study our research group has obtained metabolomics data that were composed of 136 CC and 149 normal controls (NOR). The plasma samples were detected on an ultra-performance liquid chromatography mass spectrometry (UPLC/MS) platform, which was a reliable technique in clinical study and has been applied to clinical trials, at positive and negative ion detection modes^37^,^38^.

The transcriptomics data from the Gene Expression Omnibus (GEO, http://www.ncbi.nlm.nih.gov/geo/) database (accession number GSE63514)^39^ comprised of 28 CC patients and 24 NOR were analyzed with Human Genome Affymetrix U133 Plus 2.0 microarrays.

### Sample collection, storage and preparation

Whole fasting blood samples (5 ml) were collected from each participant using EDTA Vacutainer Tubes. The blood samples were then centrifuged at 1000 × g for 10 min at 4 °C to collect the supernatant, and the collected plasma was then stored at −80 °C in a refrigerator until further analysis.

Plasma samples were thawed in a 4 °C refrigerator for 50 min after they were collected from a refrigerator set at −80 °C. Before sample preparation, quality control (QC) samples were prepared by mixing equal volumes of supernatant from all samples. After vortexing for 10 sec, the plasma was centrifuged at 4000 × g for 10 min at 4 °C. The supernatant (200 μL) was then transferred into a 2 ml centrifuge tube, mixed with 600 μL acetonitrile and vortexed for 1 min. The mixture was placed in ice water for 15 min and centrifuged at 12000 × g for 15 min at 4 °C. The supernatant (200 μL) was again transferred into a 2 ml centrifuge tube and dried in a vacuum rotary dryer. The residue was dissolved in 100 μL of acetonitrile/water (1:3, v/v), vortexed for 5 min and centrifuged at 12000 × g for 15 min at 4 °C. The extracted supernatant (90 μL) was then injected into a sample vial for LC/MS analysis.

### Metabolic profiling analysis

The metabolic profiling analysis was conducted on an UPLC system (Waters, Milford, USA) that was coupled to a 6520 series accurate quadrupole time-of-flight mass spectrometer (Q-TOF MS) system (Agilent, Santa Clara, CA, USA). The sample (10 μl) was injected into a 2.1 × 100 mm (1.7 μm) ACQUITY UPLC BEH C18 column (Waters, Milford, MA) for UPLC/MS analysis. The column oven was set at 40 °C, and the sample manager temperature was maintained at 4 °C. The mobile phase consisted of acetonitrile containing 0.1% formic acid for canal A and deionized water containing 0.1% formic acid for canal B was set at a flow rate of 0.3 ml/min. A linear gradient for elution was set as follows: 1% A for 0–0.5 min; 1–15% A for 0.5–4.0 min; 15–55% A for 4.0–4.5 min; 55–90% A for 4.5–11.5 min; 90–99% A for 11.5–12.0 min; and 99% A for 12.0–15.0 min. After the analytical run, the mobile phase was returned to 1% A in 0.1 min and equilibrated at 1% A for 1 min.

The MS acquisition and MS/MS identification were both performed in the positive-ion (ESI+) and negative-ion (ESI-) modes. The parameters for the MS acquisition were as follow: the MS capillary voltages were set at 4.0 kV in the ESI+ mode and 3.5 kV in the ESI- mode. The desolvation temperature was 330 °C, and the flow rate of the desolvation gas was 10 L/min. Centroid data were collected in the full scan mode from 77 to 1000 m/z in the positive mode and from 70 to 1100 m/z in the negative mode with a scan rate of 1.5 spectra/s.

One blank sample (25% acetonitrile) and one QC sample were run for every 15 samples to ensure the detection stability and replicability of the samples. The samples were randomized before analysis to avoid differences caused by the injection sequence.

### Data processing

The raw metabolomics data files were converted to mzdata format files by the export wizards of Agilent MassHunter Qualitative Analysis Software. Then, the files were imported to the xcms package in R language for preprocessing, which included: filtration and peak identification, matching peaks across samples, retention time correction, and filling in missing peak data^40^. The algorithm for the peak detection was findPeaks.centWave (method=“centWave”). The peak width range was set from 5 to 20 (peakwidth = c(5,20)). The bandwidth was set at 10 sec (bw = 10). Other parameters of the xcms package were set to default values. The xcmsSet object was then imported to the CAMERA package for annotation of isotope peaks, adducts and fragments in peak lists^41^. All parameters of the CAMERA packages were the default values.

### Statistical analysis

Autoscaling was used on metabolomics data before multivariate analysis, in which the centered metabolite intensity was divided by the standard deviation^42^. Unsupervised principal component analysis (PCA) was first used to detect the stability of analyses^43^. Supervised partial least-squares discriminant analysis (PLS-DA) was applied to reveal the global metabolic differences of CC and NOR^43^. Seven-fold cross-validation analyzed in SIMCA-p v11.5 (Umetrics AB, Umea, Sweden) was used for PLS-DA to evaluate the stability and credibility^44^.

The univariate nonparametric Kruskal–Wallis rank sum test and multivariate PLS-DA were performed for all metabolites^45^,^46^. In order to decrease the false discovery rate (FDR) for biomarker selection, local FDR (lfdr) based on P value was calculated to adjust the multiple comparisons^47^. The potential biomarkers were selected as univariate lfdr < 0.05 and multivariate VIP > 1. Furthermore, hierarchical cluster analysis (HCA) was conducted to detect the classification ability and concentration levels of our selected metabolites^48^. In order to evaluate the differential performance of metabolites between two groups, the area under the receiver operating characteristics (AUC) values on the test set were presented^49^.

Pathway information was extracted from Kyoto Encyclopedia of Genes and Genomes (KEGG)^50^. Kruskal–Wallis rank sum test was used to select genes from the pathways we had chosen. Lfdr values were also estimated. PantherDB analysis was performed online (pantherdb.org) to explore the molecular functions of differentially expressed genes (DEGs)^51^. All the other statistical analyses and visualizations were performed using the R platform.

### Biomarker identification and selection for cervical cancer diagnosis

The accurate masses of differential ions were used to search online databases (METLIN^52^, HMDB^53^ and MassBank^54^). The MS/MS spectra of metabolites were compared with the corresponding spectra in the online databases and confirmed with reference standards if necessary. the detailed procedures for biomarker identification were similar to those in our previous study and we also appended it in the Supplementary information 55. In order to select proper metabolites for cervical cancer diagnosis, we first clustered metabolites into co-regulated groups using Pearson correlation coefficient based on their metabolomic profiling^56^. Then the metabolites with the maximal AUC values in each cluster were selected as candidate biomarkers. The AUC value, sensitivity (SE) and specificity (SP) of the combination of these biomarkers were calculated to describe the diagnostic accuracy between CC and NOR.

### Joint analysis of metabolites and genes

Metabolites and genes in the same pathways were always dysregulated together, so we used a pathway-based approach and integrated different levels of omics in the biological process. Pathway and network analyses were firstly performed to further interpret statistical results within a biological context and explore differential metabolites and genes in cancer metabolism. Pathway analysis was conducted with MetaboAnalyst 3.0^57^. After uploaded our differential metabolites on Metaboanalyst, the metabolites were then mapped to KEGG metabolic pathways for pathway enrichment analysis and pathway topology analysis. Pathways with P < 0.1 were considered as significantly enriched pathways. Fully connected networks of metabolites and genes were then built and analyzed in Metscape^58^, which was a plug-in for Cytoscape^59^. Metscape could help us build the network of metabolites and genes, trace the connections between them, and visualize compound networks.

## Additional Information

**How to cite this article:** Yang, K. *et al*. A Comprehensive Analysis of Metabolomics and Transcriptomics in Cervical Cancer. *Sci. Rep.* **7**, 43353; doi: 10.1038/srep43353 (2017).

**Publisher's note:** Springer Nature remains neutral with regard to jurisdictional claims in published maps and institutional affiliations.

## Supplementary Material

Supplementary Information

## Figures and Tables

**Table 1:** The demographic and clinical characteristics of CC and NOR in the training and test samples.

Characteristics	Training set		Test set		GSE63514	
	CC	NOR	CC	NOR	CC	NOR
Number of subjects	70	80	66	69	28	24
Age (median, range)	48.62 (32.82–66.73)	52.00 (41.00–69.00)	49.84 (40.94–66.12)	54.00 (41.00–68.00)	44.5	28.5
Weight (median, range)	59.50 (43.00–86.00)	—	59.00 (44.00–86.00)	—	—	—
Menopause (pre/post/Undocumented)	42/25/3	—	29/32/5	—	—	—
**SCC-Ag**						
<1.5	29	—	24	—	—	—
> = 1.5	39	—	39	—	—	—
Undocumented	2	—	3	—	—	—
**FIGO stage**						
I	26	—	21	—	—	—
II	32	—	32	—	—	—
	0		1		—	—
Undocumented	12	—	12	—	—	—
**Lymphatic metastasis**						
No	39	—	39	—	—	—
Yes	11	—	8	—	—	—
Undocumented	20	—	19	—	—	—
**Histological type**						
Squamous carcinoma	54	—	54	—	—	—
Other	4	—	3	—	—	—
Undocumented	12	—	9	—	—	—
**Histology differentiation**						
Well differentiated	0	—	1	—	—	—
Moderately differentiated	15	—	21	—	—	—
Poorly differentiated	27	—	29	—	—	—
Undocumented	28	—	15	—	—	—

**Table 2:** AUC, SE and SP of 5 biomarkers and the combination of these biomarkers.

Biomarker	AUC	SE	SP
Bilirubin	0.88	0.91	0.71
LysoPC (17:0)	0.94	0.94	0.86
N-oleoyl threonine	0.85	0.83	0.79
12-Hydroxydodecanoic acid	0.92	0.94	0.79
Tetracosahexaenoic acid	0.82	0.75	0.76
Combination	0.99	0.98	0.99
