# Supplementary material for: A Comprehensive Analysis of Metabolomics and Transcriptomics in Cervical Cancer
Source: Sci Rep. 2017 Feb 22;7:43353. doi: 10.1038/srep43353 (PMC5320559; doi:10.1038/srep43353)
Supplement: Supplementary Information [file srep43353-s1.pdf]

# A Comprehensive Analysis of Metabolomics and Transcriptomics in Cervical Cancer

Kai Yang[1,†], Bairong Xia[2,†], Wenjie Wang[1], Jinlong Cheng[2], MingzhuYin[3], Hongyu Xie[1], Junnan Li[1], Libing Ma[1], Chunyan Yang[1], Ang Li[1], Xin Fan[4], Harman S Dhillon[5], Yan Hou[1,6,]*, Ge Lou[2,]* and Kang Li[1,]*

[1]Department of Epidemiology and Biostatistics, School of Public Health, Harbin Medical University, Harbin, 150086, P.R. China

[2]Department of Gynecology Oncology, the Tumor Hospital, Harbin Medical University, Harbin, 150086, P.R. China

[3]State Key Laboratory of Natural Products, Jiangsu Key Laboratory of TCM Evaluation and Translational Research Department of Complex Prescription of TCM, P.R. China Pharmaceutical University, 639 Longmian Road, Nanjing 211198, P.R. China

[4]School of Basic Medical Sciences, Heilongjiang University of Chinese Medicine, Harbin, Heilongjiang 150040, P.R. China.

[5]Harbin Medical University, Harbin, 150086, P.R. China

[6]Key Laboratory of Cardiovascular Medicine Research, Harbin Medical University, Ministry of Education, Harbin, 150086, P.R. China

[*]Address correspondence to: Y.H. (email: houyan@ems.hrbmu.edu.cn), K.L. (email: likang@ems.hrbmu.edu.cn) or G.L. (email: louge@ems.hrbmu.edu.cn)

[†]These authors contributed equally to this work.

## Strategy of metabolite identification

First, peak lists and annotation results obtained by R package XCMS[1] and CAMERA[2] were analyzed to find quasi-molecular ions and determine the corresponding molecular weights.

Second, the exact molecular weights of the monoisotopic ions were used to search the online databases, such as HMDB[3] (http://www.hmdb.ca/), METLIN[4] (http://metlin.scripps.edu/) and MassBank[5] (http://www.massbank.jp/). The mass tolerance between the measured m/z values and the exact mass of the components of interest was set within 10 ppm.

Third, a Q-TOF mass analyzer (6520, Agilent) was used to produce fragmentation patterns and structure information for biomarker candidates in the MS/MS experiments. The MS/MS fragmentation patterns of the biomarkers were compared to the spectral data of metabolites that had the same m/z in HMDB, METLIN or MassBank.

Finally, if available, confirmation with standards was performed by comparison of retention time, isotopic distribution, and fragments of reference standards (Sigma-Aldrich) with those obtained in real samples.

Metabolite identification was classed into 4 levels based on the Metabolomics Standard Initiative[6].

The first level (level 1): Metabolites are verified by reference standards.

The second level (level 2): Metabolites are identified based on accurate mass data, retention time, experimental MS/MS spectra, and library MS/MS spectra.

The third level (level 3): Metabolites are identified based on accurate mass data, retention time, experimental MS/MS spectra, and knowledge of characteristic MS/MS ions spectra.

The fourth level (level 4): Metabolites are speculated based on accurate mass data, retention time, and biological relevance. Metabolites in this level would be further validated in the future with the improvement of library spectra and availability of reference standards

**References**

1 Smith, C. A., Want, E. J., O'Maille, G., Abagyan, R. & Siuzdak, G. XCMS: processing mass spectrometry data for metabolite profiling using nonlinear peak

alignment, matching, and identification. *Analytical chemistry* **78**, 779-787, doi:10.1021/ac051437y (2006).

2 Kuhl, C., Tautenhahn, R., Bottcher, C., Larson, T. R. & Neumann, S. CAMERA: an integrated strategy for compound spectra extraction and annotation of liquid chromatography/mass spectrometry data sets. *Analytical chemistry* **84**, 283-289, doi:10.1021/ac202450g (2012).

3 Wishart, D. S. *et al.* HMDB: a knowledgebase for the human metabolome. *Nucleic acids research* **37**, D603-610, doi:10.1093/nar/gkn810 (2009).

4 Smith, C. A. *et al.* METLIN: a metabolite mass spectral database. *Ther Drug Monit* **27**, 747-751 (2005).

5 Horai, H. *et al.* MassBank: a public repository for sharing mass spectral data for life sciences. *J Mass Spectrom* **45**, 703-714, doi:10.1002/jms.1777 (2010).

6 Sumner, L. W. *et al.* Proposed minimum reporting standards for chemical analysis Chemical Analysis Working Group (CAWG) Metabolomics Standards Initiative (MSI). *Metabolomics* **3**, 211-221, doi:10.1007/s11306-007-0082-2 (2007).

**Supplementary Table S1: The detailed statistical and database information of metabolites in this study.**

| No. | mode | m/z | RT(min) | PPM | Metabolite | HMDB/METLIN | PubChem | Level[#] | lfdr | VIP | FC | AUC |
|---|---|---|---|---|---|---|---|---|---|---|---|---|
| 1 | ESI+ | 146.1167 | 4.43 | 5.90 | 4-Trimethylammonio-butanoic acid | HMDB01161 | 134 | 3 | 5.01E-04 | 1.06 | 0.70 | 0.69 |
| 2 | ESI+ | 147.1143 | 1.55 | 9.95 | L-Lysine | HMDB00182 | 5962 | 1 | 4.66E-06 | 1.18 | 0.53 | 0.75 |
| 3 | ESI+ | 169.0355 | 2.13 | 0.60 | Uric acid | HMDB00289 | 1175 | 1 | 1.43E-03 | 1.02 | 0.77 | 0.51 |
| 4 | ESI+ | 180.0654 | 5.62 | 0.72 | Hippuric acid | HMDB00714 | 464 | 1 | 6.18E-05 | 1.09 | 0.40 | 0.65 |
| 5 | ESI+ | 229.2159 | 12.79 | 1.21 | Myristic acid | HMDB00806 | 11005 | 3 | 1.42E-07 | 1.31 | 0.64 | 0.76 |
| 6 | ESI+ | 263.2369 | 13.31 | 8.87 | Palmitaldehyde | HMDB01551 | 984 | 3 | 7.70E-06 | 1.39 | 0.71 | 0.75 |
| 7 | ESI+ | 274.2742 | 6.69 | 0.19 | Hexadecasphinganine | 392316* | 656816 | 3 | 4.76E-08 | 1.53 | 0.68 | 0.79 |
| 8 | ESI+ | 277.2151 | 11.65 | 4.64 | palmitoleic acid | HMDB03229 | 445638 | 3 | 2.80E-05 | 1.29 | 0.59 | 0.77 |
| 9 | ESI+ | 302.3051 | 7.49 | 0.97 | Sphinganine | HMDB00269 | 91486 | 4 | 4.66E-06 | 1.29 | 0.72 | 0.73 |
| 10 | ESI+ | 328.3188 | 13.76 | 6.75 | N,N-Dimethyl-sphingosine | HMDB13645 | 5282309 | 4 | 2.14E-14 | 1.91 | 0.60 | 0.90 |
| 11 | ESI+ | 329.2476 | 12.88 | 0.14 | Docosahexaenoic acid | HMDB02183 | 445580 | 3 | 1.79E-08 | 1.63 | 0.57 | 0.79 |
| 12 | ESI+ | 353.2658 | 13.39 | 7.81 | MG(0:0/18:3/0:0) | HMDB11539 | 11646044 | 4 | 3.43E-04 | 1.17 | 0.66 | 0.71 |
| 13 | ESI+ | 357.241 | 11.88 | 2.81 | Tetrahydrodeoxy-corticosterone | HMDB00879 | 101771 | 4 | 2.11E-10 | 1.77 | 0.54 | 0.76 |

| | | | | | | | | | | | | |
|---|---|---|---|---|---|---|---|---|---|---|---|---|
| 14 | ESI+ | 361.1999 | 6.38 | 2.97 | Aldosterone | HMDB00037 | 24758425 | 4 | 4.66E-06 | 1.56 | 1.32 | 0.73 |
| 15 | ESI+ | 384.3076 | 6.73 | 8.45 | N-oleoyl threonine | 75490* | 52922063 | 3 | 1.77E-10 | 1.74 | 0.51 | 0.85 |
| 16 | ESI+ | 466.315 | 6.49 | 2.80 | Glycocholic Acid | HMDB00138 | 23617285 | 1 | 2.03E-05 | 1.04 | 2.38 | 0.69 |
| 17 | ESI+ | 468.3085 | 8.09 | 0.11 | LysoPC(14:0) | HMDB10379 | 460604 | 3 | 1.69E-14 | 1.81 | 0.45 | 0.86 |
| 18 | ESI+ | 472.302 | 7.00 | 2.81 | Chenodeoxy-glycocholic acid | HMDB06898 | 53477907 | 2 | 1.79E-08 | 1.34 | 2.45 | 0.76 |
| 19 | ESI+ | 480.3428 | 10.31 | 4.20 | LysoPC(P-16:0) | HMDB10407 | 10917802 | 3 | 1.06E-16 | 2.09 | 0.59 | 0.92 |
| 20 | ESI+ | 482.3241 | 8.87 | 0.02 | LysoPC(15:0) | HMDB10381 | 24779458 | 3 | 1.06E-16 | 2.12 | 0.49 | 0.92 |
| 21 | ESI+ | 496.3407 | 9.80 | 1.84 | LysoPC(16:0) | HMDB10382 | 460602 | 1 | 7.89E-20 | 2.33 | 0.68 | 0.94 |
| 22 | ESI+ | 506.3587 | 10.77 | 3.62 | CerP(d18:1/8:0) | 83734* | 10864091 | 3 | 3.38E-18 | 2.22 | 0.51 | 0.94 |
| 23 | ESI+ | 510.3557 | 10.81 | 0.62 | LysoPC(17:0) | HMDB12108 | 24779463 | 3 | 3.38E-18 | 2.18 | 0.51 | 0.94 |
| 24 | ESI+ | 518.3242 | 8.17 | 0.11 | LysoPC(18:3) | HMDB10387 | 52924045 | 3 | 7.25E-10 | 1.47 | 0.61 | 0.83 |
| 25 | ESI+ | 522.3567 | 10.25 | 2.52 | LysoPC(18:1) | HMDB02815 | 16081932 | 3 | 1.06E-13 | 1.92 | 0.67 | 0.88 |
| 26 | ESI+ | 524.372 | 11.88 | 1.80 | LysoPC(18:0) | HMDB10384 | 497299 | 1 | 6.33E-18 | 2.23 | 0.60 | 0.94 |
| 27 | ESI+ | 536.3714 | 11.19 | 0.59 | LysoPE(22:1) | HMDB11491 | 53480940 | 3 | 1.06E-17 | 2.15 | 0.54 | 0.93 |

| | | | | | | | | | | | | | |
|---|---|---|---|---|---|---|---|---|---|---|---|---|---|
| 28 | ESI+ | 544.3375 | 10.23 | 4.07 | LysoPC(20:4) | HMDB10395 | 24779476 | 3 | 2.16E-07 | 1.39 | 0.68 | 0.79 |
| 29 | ESI+ | 546.3543 | 11.88 | 2.05 | LysoPC(20:3) | HMDB10393 | 53480467 | 3 | 3.88E-09 | 1.55 | 0.64 | 0.83 |
| 30 | ESI+ | 562.4223 | 12.92 | 1.45 | CerP(d18:1/12:0) | HMDB10699 | 5283580 | 3 | 1.09E-12 | 1.79 | 0.56 | 0.88 |
| 31 | ESI+ | 568.3401 | 8.93 | 0.57 | LysoPC(22:6) | HMDB10404 | 10415542 | 3 | 4.66E-06 | 1.41 | 0.75 | 0.83 |
| 32 | ESI+ | 570.3553 | 9.38 | 0.18 | LysoPC(22:5) | HMDB10402 | 53480473 | 3 | 1.85E-03 | 1.21 | 0.78 | 0.82 |
| 33 | ESI+ | 585.2709 | 6.35 | 0.26 | Bilirubin | HMDB00054 | 5280352 | 1 | 1.94E-12 | 1.67 | 0.37 | 0.88 |
| 34 | ESI+ | 777.6875 | 6.38 | 8.32 | L-Thyroxine | HMDB00248 | 5819 | 1 | 1.33E-02 | 1.1 | 1.23 | 0.68 |
| 35 | ESI- | 87.0091 | 1.87 | 3.84 | Pyruvate | HMDB00243 | 1060 | 2 | 5.43E-15 | 1.73 | 0.41 | 0.83 |
| 36 | ESI- | 105.0194 | 1.82 | 1.11 | L-Glyceric acid | HMDB06372 | 6326776 | 3 | 2.68E-08 | 1.51 | 0.59 | 0.77 |
| 37 | ESI- | 133.0144 | 1.82 | 1.01 | L-Malic acid | HMDB00156 | 222656 | 1 | 1.30E-03 | 1.10 | 0.48 | 0.60 |
| 38 | ESI- | 145.0134 | 1.88 | 5.64 | Oxoglutaric acid | HMDB00208 | 51 | 2 | 2.21E-09 | 1.46 | 0.49 | 0.81 |
| 39 | ESI- | 154.0619 | 1.64 | 1.76 | L-Histidine | HMDB00177 | 6274 | 1 | 4.62E-04 | 1.03 | 0.71 | 0.54 |
| 40 | ESI- | 174.0884 | 1.68 | 0.20 | Citrulline | HMDB00904 | 9750 | 2 | 9.53E-05 | 1.12 | 0.67 | 0.62 |
| 41 | ESI- | 187.1338 | 6.97 | 0.66 | 3-Hydroxycapric acid | HMDB02203 | 26612 | 3 | 1.99E-05 | 1.14 | 0.65 | 0.71 |

| | | | | | | | | | | | | |
|---|---|---|---|---|---|---|---|---|---|---|---|---|
| 42 | ESI- | 215.1635 | 1.68 | 8.39 | 12-Hydroxy-dodecanoic acid | HMDB02059 | 79034 | 3 | 1.47E-09 | 1.36 | 1.72 | 0.92 |
| 43 | ESI- | 249.0891 | 4.69 | 9.43 | Threoninyl-Methionine | HMDB29067 | 7020165 | 2 | 9.09E-06 | 1.23 | 1.68 | 0.77 |
| 44 | ESI- | 255.2335 | 13.97 | 2.30 | Palmitic acid | HMDB00220 | 985 | 3 | 6.54E-05 | 1.01 | 0.55 | 0.69 |
| 45 | ESI- | 263.129 | 6.58 | 0.45 | γ-CEHC | 44822* | 15887183 | 2 | 9.09E-06 | 1.21 | 0.66 | 0.56 |
| 46 | ESI- | 275.2013 | 11.66 | 1.41 | Stearidonic Acid | HMDB06547 | 5312508 | 3 | 5.26E-08 | 1.34 | 0.47 | 0.82 |
| 47 | ESI- | 277.2178 | 12.5 | 1.92 | α-Linolenic Acid | HMDB01388 | 5280934 | 2 | 1.04E-06 | 1.14 | 0.57 | 0.75 |
| 48 | ESI- | 279.2341 | 13.33 | 4.26 | Linoleic acid | HMDB00673 | 5280450 | 3 | 3.67E-06 | 1.11 | 0.61 | 0.72 |
| 49 | ESI- | 281.2496 | 14.05 | 3.57 | Oleic Acid | HMDB00207 | 445639 | 3 | 2.83E-05 | 1.08 | 0.57 | 0.69 |
| 50 | ESI- | 297.2437 | 12.49 | 0.64 | 3-Oxooctadecanoic acid | HMDB10736 | 5283005 | 3 | 5.77E-07 | 1.29 | 0.69 | 0.77 |
| 51 | ESI- | 299.259 | 13.94 | 0.58 | 3R-hydroxy-octadecanoic acid | HMDB10737 | 5312838 | 3 | 1.20E-04 | 1.04 | 0.67 | 0.66 |
| 52 | ESI- | 321.2051 | 13.04 | 3.77 | N1,N12-Diacetylspermine | HMDB02172 | 132680 | 4 | 2.26E-05 | 1.04 | 0.66 | 0.68 |
| 53 | ESI- | 329.249 | 13.32 | 1.11 | Docosapentaenoic acid | HMDB06528 | 5497182 | 3 | 8.59E-06 | 1.23 | 0.54 | 0.71 |
| 54 | ESI- | 345.2051 | 12.5 | 5.87 | 21-Deoxycortisol | HMDB04030 | 222803 | 4 | 2.11E-07 | 1.33 | 0.64 | 0.76 |
| 55 | ESI- | 355.2648 | 13.6 | 1.52 | Tetracosahexaenoic acid | HMDB02007 | 11792612 | 3 | 1.04E-11 | 1.52 | 0.35 | 0.82 |

| | | | | | | | | | | | | |
|---|---|---|---|---|---|---|---|---|---|---|---|---|
| 56 | ESI- | 448.3073 | 7.00 | 1.10 | Glycoursodeoxycholic acid | HMDB00708 | 12310288 | 2 | 1.81E-09 | 1.20 | 2.68 | 0.74 |
| 57 | ESI- | 480.3088 | 9.82 | 1.60 | LysoPE(18:0) | HMDB11129 | 53480667 | 3 | 5.43E-15 | 1.42 | 0.75 | 0.88 |
| 58 | ESI- | 506.3255 | 10.28 | 0.60 | LysoPE(20:1) | HMDB11482 | 53480931 | 3 | 3.67E-13 | 1.70 | 0.70 | 0.90 |
| 59 | ESI- | 566.5492 | 10.28 | 4.51 | Cer(d18:0/18:0) | HMDB11761 | 5283573 | 3 | 1.39E-13 | 1.80 | 0.58 | 0.92 |
| 60 | ESI- | 588.5372 | 9.04 | 1.85 | Cer(d18:2/20:1) | 83726* | 52931121 | 3 | 4.62E-04 | 1.03 | 0.70 | 0.82 |
| 61 | ESI- | 590.5532 | 9.69 | 2.40 | Cer(d18:2/20:0) | 83725* | 52931120 | 3 | 9.09E-06 | 1.13 | 0.64 | 0.77 |
| 62 | ESI- | 608.3571 | 9.05 | 0.33 | PGPC | 45374* | 46907872 | 3 | 9.09E-06 | 1.13 | 0.70 | 0.63 |

*Referred database: METLIN ID.

# Metabolite identification was classed into 4 levels based on the Metabolomics Standard Initiative: 1. Metabolites are verified by reference standards. 2. Metabolites are identified based on accurate mass data, retention time, experimental MS/MS spectra, and library MS/MS spectra. 3. Metabolites are identified based on accurate mass data, retention time, experimental MS/MS spectra, and knowledge of characteristic MS/MS ions spectra. 4. Metabolites are speculated based on accurate mass data, retention time, and biological relevance.

**Supplementary Table S2: Metabolic pathway of metabolites in this study.**

| **Pathway** | **Metabolites** | **biofunction** |
|---|---|---|
| Citrate cycle | Pyruvate, Oxoglutaric acid, L-Malic acid | Energy source,<br>Pyruvate metabolism, amino acid metabolism |
| Glycerophospholipid metabolism | LysoPC(14:0), LysoPC(15:0), LysoPC(16:0), LysoPC(P-16:0), LysoPC(17:0), LysoPC(18:0), LysoPC(18:1), LysoPC(18:3), LysoPC(20:3), LysoPC(20:4), LysoPC(22:5), LysoPC(22:6) | Cell signaling,<br>membrane integrity |
| Lysophospholipid metabolism | LysoPE(18:0), LysoPE(20:1) LysoPE(22:1) | Cell signaling,<br>membrane integrity |
| Sphingolipid metabolism | Sphinganine, Hexadecasphinganine, N,N-Dimethylsphingosine, Cer(d18:0/18:0), Cer(d18:2/20:0), Cer(d18:2/20:1), CerP(d18:1/12:0), CerP(d18:1/8:0) | Cell signaling,<br>membrane integrity |
| Steroid hormone biosynthesis | 21-Deoxycortisol, Aldosterone, Tetrahydrodeoxycorticosterone | Cell signaling |
| Bile acid biosynthesis | Glycocholic Acid, Glycoursodeoxycholic acid, Chenodeoxyglycocholic acid | Cell signaling, fuel and energy source |
| Lysine metabolism | L-Lysine, 4-Trimethylammoniobutanoic acid | Aminoacyl-tRNA biosynthesis , amino acid metabolism |
| Phenylalanine metabolism | Hippuric acid | Aminoacyl-tRNA biosynthesis, amino acid metabolism |
| Tyrosine metabolism | L-Thyroxine | Oxidative |

| | | |
|---|---|---|
| | | phosphorylation |
| Histidine metabolism | L-Histidine | Aminoacyl-tRNA biosynthesis, amino acid metabolism |
| Arginine biosynthesis | Citrulline | amino acid metabolism |
| Glycine, serine and threonine metabolism | L-Glyceric acid | Protein synthesis, amino acid metabolism |
| Protein catabolism | Threoninyl-Methionine, N-oleoyl threonine | Not Available |
| Vitamin E metabolism | γ-CEHC | Antioxidant activity, Natriuresis |
| Purine metabolism | Uric acid | Not Available |
| Porphyrin and chlorophyll metabolism | Bilirubin | Heme degradation |
| Glutathione metabolism | 12-Hydroxydodecanoic acid | Cell signaling, membrane integrity, fuel and energy source |
| Fatty acid degradation | 3-Hydroxycapric acid, Palmitaldehyde | Cell signaling, membrane integrity, fuel and energy source |
| Fatty acid metabolism | 3-Oxooctadecanoic acid, Tetracosahexaenoic acid | Cell signaling, membrane integrity, fuel and energy source |
| Fatty acid biosynthesis | 3R-hydroxy-octadecanoic acid, palmitoleic acid, Myristic acid | Cell signaling, membrane integrity, fuel |

| | | |
|---|---|---|
| | | and energy source |
| Biosynthesis of unsaturated fatty acids | Oleic Acid, Palmitic acid, Docosahexaenoic acid, docosapentaenoic acid, Linoleic acid | Cell signaling, membrane integrity, fuel and energy source |
| alpha-Linolenic acid metabolism | Stearidonic Acid, α-Linolenic Acid | Cell signaling, membrane integrity, fuel and energy source |
| Other | N1,N12-Diacetylspermine, MG(0:0/18:3/0:0), PGPC | |

**Supplementary Table S3: the clustering results of all metabolites based on their metabolic profiling.**

| Cluster | Metabolites |
|---|---|
| Cluster1 | 3R-hydroxy-octadecanoic acid, L-Histidine, L-Lysine, 4-Trimethylammoniobutanoic acid, L-Glyceric acid, Hippuric acid, Citrulline, L-Malic acid, Oxoglutaric acid, γ-CEHC, Bilirubin, PGPC |
| Cluster2 | α-Linolenic Acid, Docosapentaenoic acid, Docosahexaenoic acid, Linoleic acid, Palmitic acid, Oleic Acid, Myristic acid, Tetracosahexaenoic acid, 3-Oxooctadecanoic acid, Palmitaldehyde, 3-Hydroxycapric acid, 21-Deoxycortisol, N1,N12-Diacetylspermine |
| Cluster3 | Stearidonic Acid, palmitoleic acid, LysoPC(14:0), LysoPC(15:0), LysoPC(16:0), LysoPC(P-16:0), LysoPC(17:0), LysoPC(18:0), LysoPC(18:1), LysoPC(18:3), LysoPC(22:6), LysoPC(22:5), LysoPE(18:0), LysoPE(20:1), LysoPE(22:1), CerP(d18:1/8:0), CerP(d18:1/12:0), Cer(d18:0/18:0), Cer(d18:2/20:0), Cer(d18:2/20:1), N,N-Dimethylsphingosine, Sphinganine, C16 Sphinganine |
| Cluster4 | LysoPC(20:3), LysoPC(20:4), Tetrahydrodeoxycorticosterone, N-oleoyl threonine, Pyruvate, Uric acid, MG(0:0/18:3/0:0) |
| Cluster5 | Glycocholic Acid, Chenodeoxyglycocholic acid, Glycoursodeoxycholic acid, Aldosterone, L-Thyroxine, Threoninyl-Methionine, 12-Hydroxydodecanoic acid |

**Supplementary Table S4: Result from pathway analysis of the 62 metabolites conducted in metaboAnalyst.**

| Pathway | Total | Hits | Raw P | FDR | Impact |
|---|---|---|---|---|---|
| Fatty acid biosynthesis | 49 | 4 | 0.0019 | 0.1509 | 0 |
| Glyoxylate and dicarboxylate metabolism | 50 | 3 | 0.0174 | 0.5422 | 0.0571 |
| Citrate cycle (TCA cycle) | 20 | 2 | 0.0203 | 0.5422 | 0.1294 |
| Lysine biosynthesis | 32 | 2 | 0.0489 | 0.9354 | 0.0999 |
| Histidine metabolism | 44 | 2 | 0.0859 | 0.9354 | 0.1399 |
| Lysine degradation | 47 | 2 | 0.0962 | 0.9354 | 0.1523 |
| Steroid hormone biosynthesis | 99 | 3 | 0.0968 | 0.9354 | 0.0284 |
| Fatty acid metabolism | 50 | 2 | 0.1069 | 0.9354 | 0.0448 |
| D-Glutamine and D-glutamate metabolism | 11 | 1 | 0.1169 | 0.9354 | 0 |
| Biotin metabolism | 11 | 1 | 0.1169 | 0.9354 | 0 |
| Linoleic acid metabolism | 15 | 1 | 0.1561 | 1 | 0.6563 |
| Aminoacyl-tRNA biosynthesis | 75 | 2 | 0.2048 | 1 | 0.0563 |
| Alanine, aspartate and glutamate metabolism | 24 | 1 | 0.2382 | 1 | 0 |
| Sphingolipid metabolism | 25 | 1 | 0.2468 | 1 | 0.1402 |
| Fatty acid elongation in mitochondria | 27 | 1 | 0.2638 | 1 | 0 |
| beta-Alanine metabolism | 28 | 1 | 0.2722 | 1 | 0 |
| alpha-Linolenic acid metabolism | 29 | 1 | 0.2804 | 1 | 0.2034 |
| Pyruvate metabolism | 32 | 1 | 0.3047 | 1 | 0 |
| Vitamin B6 metabolism | 32 | 1 | 0.3047 | 1 | 0.0191 |
| Glycerolipid metabolism | 32 | 1 | 0.3047 | 1 | 0.0206 |

| | | | | | |
|---|---|---|---|---|---|
| Pentose phosphate pathway | 32 | 1 | 0.3047 | 1 | 0.0218 |
| Nitrogen metabolism | 39 | 1 | 0.3582 | 1 | 0 |
| Glycerophospholipid metabolism | 39 | 1 | 0.3582 | 1 | 0.0032 |
| Butanoate metabolism | 40 | 1 | 0.3655 | 1 | 0 |
| Ascorbate and aldarate metabolism | 45 | 1 | 0.4009 | 1 | 0 |
| Phenylalanine metabolism | 45 | 1 | 0.4009 | 1 | 0.0315 |
| Primary bile acid biosynthesis | 47 | 1 | 0.4145 | 1 | 0.0085 |
| Glycine, serine and threonine metabolism | 48 | 1 | 0.4212 | 1 | 0.0005 |
| Tyrosine metabolism | 76 | 1 | 0.5815 | 1 | 0 |
| Arginine and proline metabolism | 77 | 1 | 0.5863 | 1 | 0.02 |
| Purine metabolism | 92 | 1 | 0.6529 | 1 | 0.0097 |

**Supplementary Table S5: The detailed statistical information of differentially expressed genes in this study.**

| Gene | lfdr | FC | Gene | lfdr | FC | Gene | lfdr | FC |
|---|---|---|---|---|---|---|---|---|
| HNMT | 3.94E-09 | 0.80 | COMT | 4.50E-04 | 0.90 | DLST | 7.00E-03 | 1.22 |
| CYP3A4 | 1.34E-08 | 0.79 | COLGALT1 | 4.50E-04 | 1.09 | SULT1E1 | 7.00E-03 | 0.78 |
| UGT2B28 | 2.38E-08 | 0.66 | AKR1C4 | 9.07E-04 | 1.03 | UGT2A3 | 7.00E-03 | 0.97 |
| WHSC1 | 8.48E-08 | 1.18 | ALDH3B1 | 9.07E-04 | 0.85 | GLYCTK | 7.09E-03 | 0.84 |
| DDC | 1.17E-07 | 0.90 | HSD17B8 | 9.07E-04 | 0.90 | GRHPR | 1.06E-02 | 0.97 |
| BBOX1 | 1.17E-07 | 0.53 | SHMT2 | 9.07E-04 | 1.06 | IDH3G | 1.06E-02 | 0.93 |
| HSD11B1 | 1.17E-07 | 1.75 | HSD17B1 | 9.07E-04 | 0.90 | HSD17B2 | 1.06E-02 | 0.71 |
| UGT2B15 | 2.76E-07 | 0.63 | ACSL4 | 1.14E-03 | 0.80 | ACSBG1 | 1.06E-02 | 0.98 |
| SDHB | 2.76E-07 | 1.25 | GCDH | 1.14E-03 | 0.86 | HAL | 1.06E-02 | 0.56 |
| STS | 1.11E-06 | 0.93 | MDH2 | 1.14E-03 | 0.90 | PCCA | 1.06E-02 | 0.97 |
| SULT2B1 | 1.39E-06 | 0.66 | GLUL | 1.14E-03 | 0.81 | KMT2B | 1.06E-02 | 0.97 |
| KMT2A | 1.39E-06 | 0.93 | ALDH3B2 | 1.30E-03 | 0.78 | CYP11B1 | 1.22E-02 | 0.98 |
| SUV39H2 | 2.58E-06 | 1.28 | ALDH1B1 | 1.30E-03 | 0.85 | SUCLG1 | 1.22E-02 | 0.97 |
| CYP19A1 | 2.58E-06 | 0.95 | ACLY | 1.30E-03 | 1.39 | UGT2B4 | 1.28E-02 | 0.98 |
| CYP11A1 | 2.58E-06 | 0.86 | AKR1C3 | 1.31E-03 | 0.74 | SDHD | 1.35E-02 | 0.99 |
| CYP3A5 | 2.58E-06 | 0.61 | HSD17B12 | 1.61E-03 | 0.96 | OGDH | 1.74E-02 | 0.90 |
| UGT2A1 | 2.58E-06 | 0.75 | ALDH2 | 1.61E-03 | 0.92 | ACSL1 | 1.74E-02 | 0.95 |
| UGT2A2 | 2.58E-06 | 0.75 | SRD5A1 | 1.61E-03 | 0.92 | HSD3B1 | 1.74E-02 | 0.98 |
| HYKK | 1.28E-05 | 0.89 | UGT2B17 | 1.61E-03 | 0.65 | ALDH7A1 | 1.74E-02 | 0.92 |
| CYP1A2 | 2.63E-05 | 0.84 | DOT1L | 1.61E-03 | 1.25 | IDH2 | 1.74E-02 | 0.93 |

| | | | | | | | | |
|---|---|---|---|---|---|---|---|---|
| SUV39H1 | 3.45E-05 | 1.16 | UGT1A10 | 2.72E-03 | 1.05 | FASN | 1.74E-02 | 0.98 |
| OLAH | 3.45E-05 | 0.90 | UGT1A3 | 2.72E-03 | 1.05 | PGP | 1.74E-02 | 1.10 |
| COLGALT2 | 3.45E-05 | 0.88 | UGT1A4 | 2.72E-03 | 1.05 | CAMKMT | 1.74E-02 | 1.11 |
| PLOD2 | 3.46E-05 | 1.90 | UGT1A5 | 2.72E-03 | 1.05 | SETDB2 | 1.74E-02 | 1.10 |
| EHMT2 | 3.46E-05 | 0.91 | UGT1A7 | 2.72E-03 | 1.05 | SETD7 | 1.74E-02 | 0.96 |
| AKR1C2 | 3.46E-05 | 1.04 | UGT1A8 | 2.72E-03 | 1.05 | HSD17B6 | 1.74E-02 | 1.32 |
| KMT2D | 9.41E-05 | 0.87 | UGT1A9 | 2.72E-03 | 1.05 | CYP7A1 | 2.53E-02 | 0.98 |
| ASH1L | 9.41E-05 | 0.79 | HOGA1 | 2.72E-03 | 1.05 | PIPOX | 2.53E-02 | 1.06 |
| WHSC1L1 | 9.41E-05 | 1.13 | SRD5A2 | 2.90E-03 | 1.08 | KMT2E | 2.53E-02 | 1.05 |
| UGT1A6 | 9.41E-05 | 0.92 | ACSL6 | 3.29E-03 | 0.97 | SETD2 | 4.16E-02 | 0.87 |
| ACAT1 | 1.83E-04 | 0.96 | SRD5A3 | 3.29E-03 | 0.81 | CNDP2 | 4.16E-02 | 0.98 |
| FH | 1.83E-04 | 0.83 | SDHA | 3.84E-03 | 1.10 | CYP17A1 | 4.40E-02 | 0.95 |
| CYP2E1 | 2.09E-04 | 1.31 | NSD1 | 3.84E-03 | 1.25 | GLDC | 4.40E-02 | 1.40 |
| ACACB | 2.37E-04 | 0.98 | MAOB | 4.33E-03 | 0.83 | PCK1 | 4.40E-02 | 1.02 |
| AOC1 | 2.37E-04 | 0.69 | MAOA | 4.33E-03 | 0.86 | DLAT | 4.40E-02 | 1.09 |
| FTCD | 2.37E-04 | 0.93 | ALDH9A1 | 4.73E-03 | 0.95 | PCCB | 4.40E-02 | 1.09 |
| SETMAR | 4.50E-04 | 1.17 | CYP11B2 | 4.73E-03 | 1.06 | HAO1 | 4.40E-02 | 0.98 |
| CYP3A7 | 4.50E-04 | 0.98 | PLOD3 | 7.00E-03 | 1.25 | ACO1 | 4.40E-02 | 1.04 |
| AGXT | 4.50E-04 | 0.97 | SETDB1 | 7.00E-03 | 1.10 | SDHC | 4.40E-02 | 1.09 |

**Supplementary Table S6: . The detailed gene name and functional information of differentially expressed genes in this study.**

| Gene Symbol | Gene Name | PANTHER GO-Slim Molecular Function |
|---|---|---|
| HNMT | Histamine N-methyltransferase | methyltransferase activity(GO:0003824) |
| CYP3A4 | Cytochrome P450 3A4 | oxidoreductase activity(GO:0003824) |
| DDC | Aromatic-L-amino-acid decarboxylase | carboxy-lyase activity(GO:0003824) |
| WHSC1 | Histone-lysine N-methyltransferase NSD2 | |
| UGT2B28 | UDP-glucuronosyltransferase 2B28 | transferase activity, transferring glycosyl groups(GO:0003824) |
| SUV39H2 | Histone-lysine N-methyltransferase SUV39H2 | |
| SULT2B1 | Sulfotransferase family cytosolic 2B member 1 | transferase activity(GO:0003824) |
| STS | Steryl-sulfatase | hydrolase activity(GO:0003824) |
| UGT2B15 | UDP-glucuronosyltransferase 2B15 | transferase activity, transferring glycosyl groups(GO:0003824) |
| SDHB | Succinate dehydrogenase [ubiquinone] iron-sulfur subunit, mitochondrial | oxidoreductase activity(GO:0003824) |
| KMT2A | Histone-lysine N-methyltransferase 2A | |
| HSD11B1 | Corticosteroid 11-beta-dehydrogenase isozyme 1 | oxidoreductase activity(GO:0003824) |
| BBOX1 | Gamma-butyrobetaine dioxygenase | oxidoreductase activity(GO:0003824) |
| CYP19A1 | Aromatase | oxidoreductase activity(GO:0003824) |
| CYP11A1 | Cholesterol side-chain cleavage enzyme, mitochondrial | oxidoreductase activity(GO:0003824) |
| CYP3A5 | Cytochrome P450 3A5 | oxidoreductase activity(GO:0003824) |
| UGT2A1 | UDP-glucuronosyltransferase 2A1 | transferase activity, transferring glycosyl groups(GO:0003824) |
| UGT2A2 | UDP-glucuronosyltransferase 2A1 | transferase activity, transferring glycosyl groups(GO:0003824) |
| HYKK | Hydroxylysine kinase | |
| CYP1A2 | Cytochrome P450 1A2 | oxidoreductase activity(GO:0003824) |
| SUV39H1 | Histone-lysine N-methyltransferase SUV39H1 | |
| OLAH | S-acyl fatty acid synthase thioesterase, medium chain | |

| | | |
|---|---|---|
| COLGALT2 | Procollagen galactosyltransferase 2 | oxidoreductase activity(GO:0003824) |
| PLOD2 | Procollagen-lysine,2-oxoglutarate 5-dioxygenase 2 | |
| EHMT2 | Histone-lysine N-methyltransferase EHMT2 | |
| AKR1C2 | Aldo-keto reductase family 1 member C2 | oxidoreductase activity(GO:0003824);transporter activity(GO:0016491) |
| KMT2D | Histone-lysine N-methyltransferase 2D | |
| ASH1L | Histone-lysine N-methyltransferase ASH1L | |
| WHSC1L1 | Histone-lysine N-methyltransferase NSD3 | |
| UGT1A6 | UDP-glucuronosyltransferase 1-6 | transferase activity, transferring glycosyl groups(GO:0003824) |
| ACAT1 | Acetyl-CoA acetyltransferase, mitochondrial | acetyltransferase activity(GO:0003824) |
| FH | Fumarate hydratase, mitochondrial | |
| CYP2E1 | Cytochrome P450 2E1 | oxidoreductase activity(GO:0003824) |
| ACACB | Acetyl-CoA carboxylase 2 | ligase activity(GO:0003824) |
| AOC1 | Amiloride-sensitive amine oxidase [copper-containing] | |
| FTCD | Formimidoyltransferase-cyclodeaminase | deaminase activity(GO:0003824);hydrolase activity(GO:0019239);transferase activity(GO:0016787) |
| SETMAR | Histone-lysine N-methyltransferase SETMAR | |
| CYP3A7 | Cytochrome P450 3A7 | oxidoreductase activity(GO:0003824) |
| AGXT | Serine--pyruvate aminotransferase | transaminase activity(GO:0003824) |
| COMT | Catechol O-methyltransferase | methyltransferase activity(GO:0003824) |
| COLGALT1 | Procollagen galactosyltransferase 1 | oxidoreductase activity(GO:0003824) |
| AKR1C4 | Aldo-keto reductase family 1 member C4 | oxidoreductase activity(GO:0003824);transporter activity(GO:0016491) |
| ALDH3B1 | Aldehyde dehydrogenase family 3 member B1 | oxidoreductase activity(GO:0003824) |
| HSD17B8 | Estradiol 17-beta-dehydrogenase 8 | oxidoreductase activity(GO:0003824) |

| | | |
|---|---|---|
| SHMT2 | Serine hydroxymethyltransferase, mitochondrial | methyltransferase activity(GO:0003824) |
| HSD17B1 | Estradiol 17-beta-dehydrogenase 1 | oxidoreductase activity(GO:0003824) |
| ACSL4 | Long-chain-fatty-acid--CoA ligase 4 | ligase activity(GO:0003824);transporter activity(GO:0016874) |
| GCDH | Glutaryl-CoA dehydrogenase, mitochondrial | nucleotide binding(GO:0005488);oxidoreductase activity(GO:0000166) |
| MDH2 | Malate dehydrogenase, mitochondrial | oxidoreductase activity(GO:0003824) |
| GLUL | Glutamine synthetase | ligase activity(GO:0003824) |
| ALDH3B2 | Aldehyde dehydrogenase family 3 member B2 | oxidoreductase activity(GO:0003824) |
| ALDH1B1 | Aldehyde dehydrogenase X, mitochondrial | oxidoreductase activity(GO:0003824) |
| ACLY | ATP-citrate synthase | ligase activity(GO:0003824);lyase activity(GO:0016874);transferase activity(GO:0016829) |
| AKR1C3 | Aldo-keto reductase family 1 member C3 | oxidoreductase activity(GO:0003824);transporter activity(GO:0016491) |
| HSD17B12 | Very-long-chain 3-oxoacyl-CoA reductase | oxidoreductase activity(GO:0003824) |
| ALDH2 | Aldehyde dehydrogenase, mitochondrial | oxidoreductase activity(GO:0003824) |
| SRD5A1 | 3-oxo-5-alpha-steroid 4-dehydrogenase 1 | oxidoreductase activity(GO:0003824) |
| UGT2B17 | UDP-glucuronosyltransferase 2B17 | transferase activity, transferring glycosyl groups(GO:0003824) |
| DOT1L | Histone-lysine N-methyltransferase, H3 lysine-79 specific | methyltransferase activity(GO:0003824) |
| UGT1A10 | UDP-glucuronosyltransferase 1-10 | transferase activity, transferring glycosyl groups(GO:0003824) |
| UGT1A3 | UDP-glucuronosyltransferase 1-3 | transferase activity, transferring glycosyl groups(GO:0003824) |
| UGT1A4 | UDP-glucuronosyltransferase 1-4 | transferase activity, transferring glycosyl groups(GO:0003824) |
| UGT1A5 | UDP-glucuronosyltransferase 1-5 | transferase activity, transferring glycosyl groups(GO:0003824) |
| UGT1A7 | UDP-glucuronosyltransferase 1-7 | transferase activity, transferring glycosyl groups(GO:0003824) |
| UGT1A8 | UDP-glucuronosyltransferase 1-8 | transferase activity, transferring glycosyl groups(GO:0003824) |
| UGT1A9 | UDP-glucuronosyltransferase 1-9 | transferase activity, transferring glycosyl groups(GO:0003824) |
| HOGA1 | 4-hydroxy-2-oxoglutarate aldolase, mitochondrial | lyase activity(GO:0003824) |

| | | |
|---|---|---|
| SRD5A2 | 3-oxo-5-alpha-steroid 4-dehydrogenase 2 | oxidoreductase activity(GO:0003824) |
| ACSL6 | Long-chain-fatty-acid--CoA ligase 6 | ligase activity(GO:0003824);transporter activity(GO:0016874) |
| SRD5A3 | Polyprenol reductase | |
| SDHA | Succinate dehydrogenase [ubiquinone] flavoprotein subunit, mitochondrial | oxidoreductase activity(GO:0003824) |
| NSD1 | Histone-lysine N-methyltransferase, H3 lysine-36 and H4 lysine-20 specific | |
| MAOB | Amine oxidase [flavin-containing] B | DNA-methyltransferase activity(GO:0003824);nucleic acid binding(GO:0016740);oxidoreductase activity(GO:0008168) |
| MAOA | Amine oxidase [flavin-containing] A | DNA-methyltransferase activity(GO:0003824);nucleic acid binding(GO:0016740);oxidoreductase activity(GO:0008168) |
| ALDH9A1 | 4-trimethylaminobutyraldehyde dehydrogenase | oxidoreductase activity(GO:0003824) |
| CYP11B2 | Cytochrome P450 11B2, mitochondrial | oxidoreductase activity(GO:0003824) |
| PLOD3 | Procollagen-lysine,2-oxoglutarate 5-dioxygenase 3 | |
| SETDB1 | Histone-lysine N-methyltransferase SETDB1 | |
| DLST | Dihydrolipoyllysine-residue succinyltransferase component of 2-oxoglutarate dehydrogenase complex, mitochondrial | acetyltransferase activity(GO:0003824) |
| SULT1E1 | Estrogen sulfotransferase | transferase activity(GO:0003824) |
| UGT2A3 | UDP-glucuronosyltransferase 2A3 | transferase activity, transferring glycosyl groups(GO:0003824) |
| GLYCTK | Glycerate kinase | kinase activity(GO:0003824) |
| GRHPR | Glyoxylate reductase/hydroxypyruvate reductase | oxidoreductase activity(GO:0003824) |
| IDH3G | Isocitrate dehydrogenase [NAD] subunit gamma, mitochondrial | oxidoreductase activity(GO:0003824) |
| HSD17B2 | Estradiol 17-beta-dehydrogenase 2 | oxidoreductase activity(GO:0003824) |
| ACSBG1 | Long-chain-fatty-acid--CoA ligase ACSBG1 | ligase activity(GO:0003824);transporter activity(GO:0016874) |
| HAL | Histidine ammonia-lyase | |

| | | |
|---|---|---|
| PCCA | Propionyl-CoA carboxylase alpha chain, mitochondrial | ligase activity(GO:0003824) |
| KMT2B | Histone-lysine N-methyltransferase 2B | |
| CYP11B1 | Cytochrome P450 11B1, mitochondrial | oxidoreductase activity(GO:0003824) |
| SUCLG1 | Succinyl-CoA ligase [ADP/GDP-forming] subunit alpha, mitochondrial | ligase activity(GO:0003824) |
| UGT2B4 | UDP-glucuronosyltransferase 2B4 | transferase activity, transferring glycosyl groups(GO:0003824) |
| SDHD | Succinate dehydrogenase [ubiquinone] cytochrome b small subunit, mitochondrial | oxidoreductase activity(GO:0003824) |
| OGDH | 2-oxoglutarate dehydrogenase, mitochondrial | oxidoreductase activity(GO:0003824) |
| ACSL1 | Long-chain-fatty-acid--CoA ligase 1 | ligase activity(GO:0003824);transporter activity(GO:0016874) |
| HSD3B1 | 3 beta-hydroxysteroid dehydrogenase/Delta 5--4-isomerase type 1 | hydro-lyase activity(GO:0003824);oxidoreductase activity(GO:0016829);racemase and epimerase activity(GO:0016836) |
| ALDH7A1 | Alpha-aminoadipic semialdehyde dehydrogenase | oxidoreductase activity(GO:0003824) |
| IDH2 | Isocitrate dehydrogenase [NADP], mitochondrial | |
| FASN | Fatty acid synthase | hydrolase activity, acting on ester bonds(GO:0003824);ligase activity(GO:0016787);methyltransferase activity(GO:0016788);transferase activity, transferring acyl groups(GO:0016874) |
| PGP | Phosphoglycolate phosphatase | hydrolase activity(GO:0003824) |
| CAMKMT | Calmodulin-lysine N-methyltransferase | |
| SETDB2 | Histone-lysine N-methyltransferase SETDB2 | |
| SETD7 | Histone-lysine N-methyltransferase SETD7 | kinase activity(GO:0003824) |
| HSD17B6 | 17-beta-hydroxysteroid dehydrogenase type 6 | oxidoreductase activity(GO:0003824) |
| CYP7A1 | Cholesterol 7-alpha-monooxygenase | oxidoreductase activity(GO:0003824) |

| | | |
|---|---|---|
| PIPOX | Peroxisomal sarcosine oxidase | oxidoreductase activity(GO:0003824) |
| KMT2E | Histone-lysine N-methyltransferase 2E | |
| SETD2 | Histone-lysine N-methyltransferase SETD2 | |
| CNDP2 | Cytosolic non-specific dipeptidase | deacetylase activity(GO:0003824);metallopeptidase activity(GO:0016787) |
| CYP17A1 | Steroid 17-alpha-hydroxylase/17,20 lyase | |
| GLDC | Glycine dehydrogenase (decarboxylating), mitochondrial | binding(GO:0005488);oxidoreductase activity(GO:0003824) |
| PCK1 | Phosphoenolpyruvate carboxykinase, cytosolic [GTP] | carboxy-lyase activity(GO:0003824) |
| DLAT | Dihydrolipoyllysine-residue acetyltransferase component of pyruvate dehydrogenase complex, mitochondrial | acetyltransferase activity(GO:0003824) |
| PCCB | Propionyl-CoA carboxylase beta chain, mitochondrial | ligase activity(GO:0003824) |
| HAO1 | Hydroxyacid oxidase 1 | isomerase activity(GO:0003824);ligase activity(GO:0016853);oxidoreductase activity(GO:0016874) |
| ACO1 | Cytoplasmic aconitate hydratase | hydro-lyase activity(GO:0003824) |
| SDHC | Succinate dehydrogenase cytochrome b560 subunit, mitochondrial | |

**Supplementary Table S7: KEGG pathways of differentially expressed genes in this study.**

| Pathway | genes |
|---|---|
| Fatty acid biosynthesis | ACACB, ACSBG1, ACSL1, ACSL4, ACSL6, FASN, OLAH |
| Glyoxylate and dicarboxylate metabolism | ACAT1, ACO1, AGXT, GLDC, GLUL, GLYCTK, GRHPR, HAO1, HOGA1, MDH2, PCCA, PCCB, PGP, SHMT2 |
| Citrate cycle (TCA cycle) | ACLY, ACO1, DLAT, DLST, FH, IDH2, IDH3G, MDH2, OGDH, PCK1, SDHA, SDHB, SDHC, SDHD, SUCLG1 |
| Lysine biosynthesis | ALDH7A1 |
| Histidine metabolism | ALDH1B1, ALDH2, ALDH3B1, ALDH3B2, ALDH7A1, ALDH9A1, AOC1, CNDP2, DDC, FTCD, HAL, HNMT, MAOA, MAOB |
| Lysine degradation | ACAT1, ALDH1B1, ALDH2, ALDH7A1, ALDH9A1, ASH1L, BBOX1, CAMKMT, COLGALT1, COLGALT2, DLST, DOT1L, EHMT2, GCDH, HYKK, KMT2A, KMT2B, KMT2D, KMT2E, NSD1, OGDH, PIPOX, PLOD2, PLOD3, SETD2, SETD7, SETDB1, SETDB2, SETMAR, SUV39H1, SUV39H2, WHSC1, WHSC1L1 |
| Steroid hormone biosynthesis | AKR1C2, AKR1C3, AKR1C4, COMT, CYP11A1, CYP11B1, CYP11B2, CYP17A1, CYP19A1, CYP1A2, CYP2E1, CYP3A4, CYP3A5, CYP3A7, CYP7A1, HSD11B1, HSD17B1, HSD17B12, HSD17B2, HSD17B6, HSD17B8, HSD3B1, SRD5A1, SRD5A2, SRD5A3, STS, SULT1E1, SULT2B1, UGT1A10, UGT1A3, UGT1A4, UGT1A5, UGT1A6, UGT1A7, UGT1A8, UGT1A9, UGT2A1, UGT2A2, UGT2A3, UGT2B15, UGT2B17, UGT2B28, UGT2B4 |

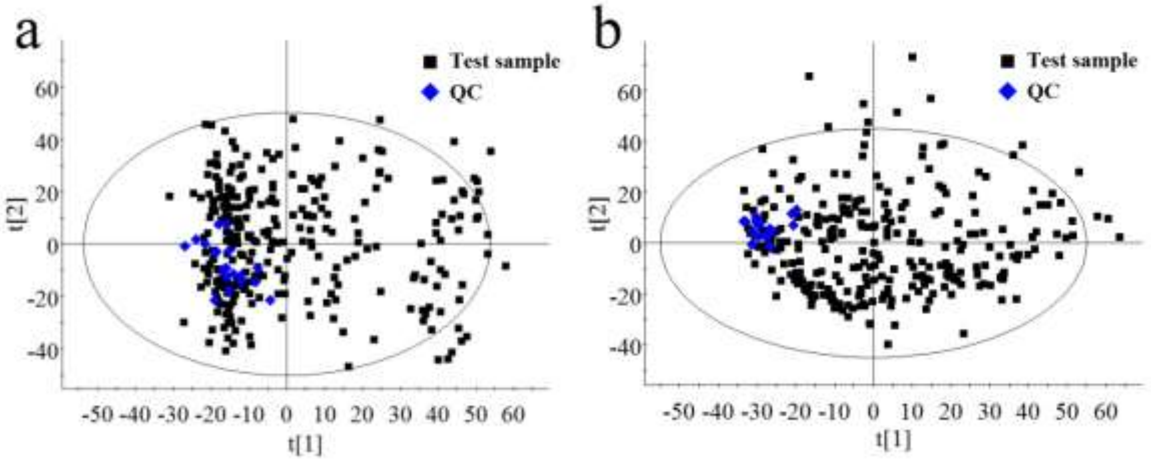


**Supplementary Figure S1:** (A)PCA score plot for test samples and QCs (quality control) in ESI+ mode. (B)PCA score plot for test samples and QCs (quality control) in ESI- mode.

a

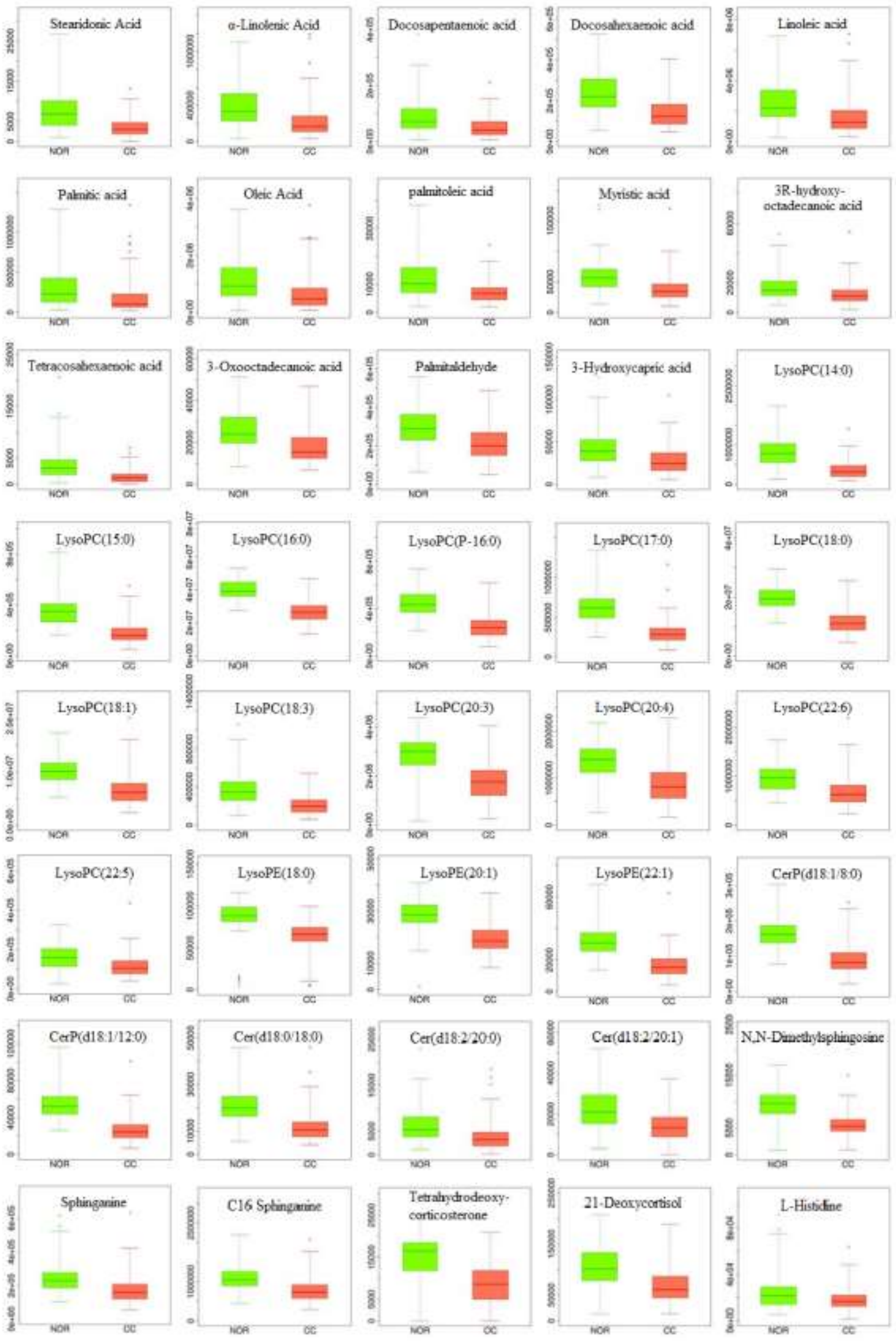
Stearidonic Acid
α-Linolenic Acid
Docosapentaenoic acid
Docosahexaenoic acid
Linoleic acid
Palmitic acid
Oleic Acid
palmitoleic acid
Myristic acid
3R-hydroxy-octadecanoic acid
Tetracosahexaenoic acid
3-Oxooctadecanoic acid
Palmitaldehyde
3-Hydroxycapric acid
LysoPC(14:0)
LysoPC(15:0)
LysoPC(16:0)
LysoPC(P-16:0)
LysoPC(17:0)
LysoPC(18:0)
LysoPC(18:1)
LysoPC(18:3)
LysoPC(20:3)
LysoPC(20:4)
LysoPC(22:6)
LysoPC(22:5)
LysoPE(18:0)
LysoPE(20:1)
LysoPE(22:1)
CerP(d18:1/8:0)
CerP(d18:1/12:0)
Cer(d18:0/18:0)
Cer(d18:2/20:0)
Cer(d18:2/20:1)
N,N-Dimethylsphingosine
Sphinganine
C16 Sphinganine
Tetrahydrodeoxy-corticosterone
21-Deoxycortisol
L-Histidine
NOR
CC

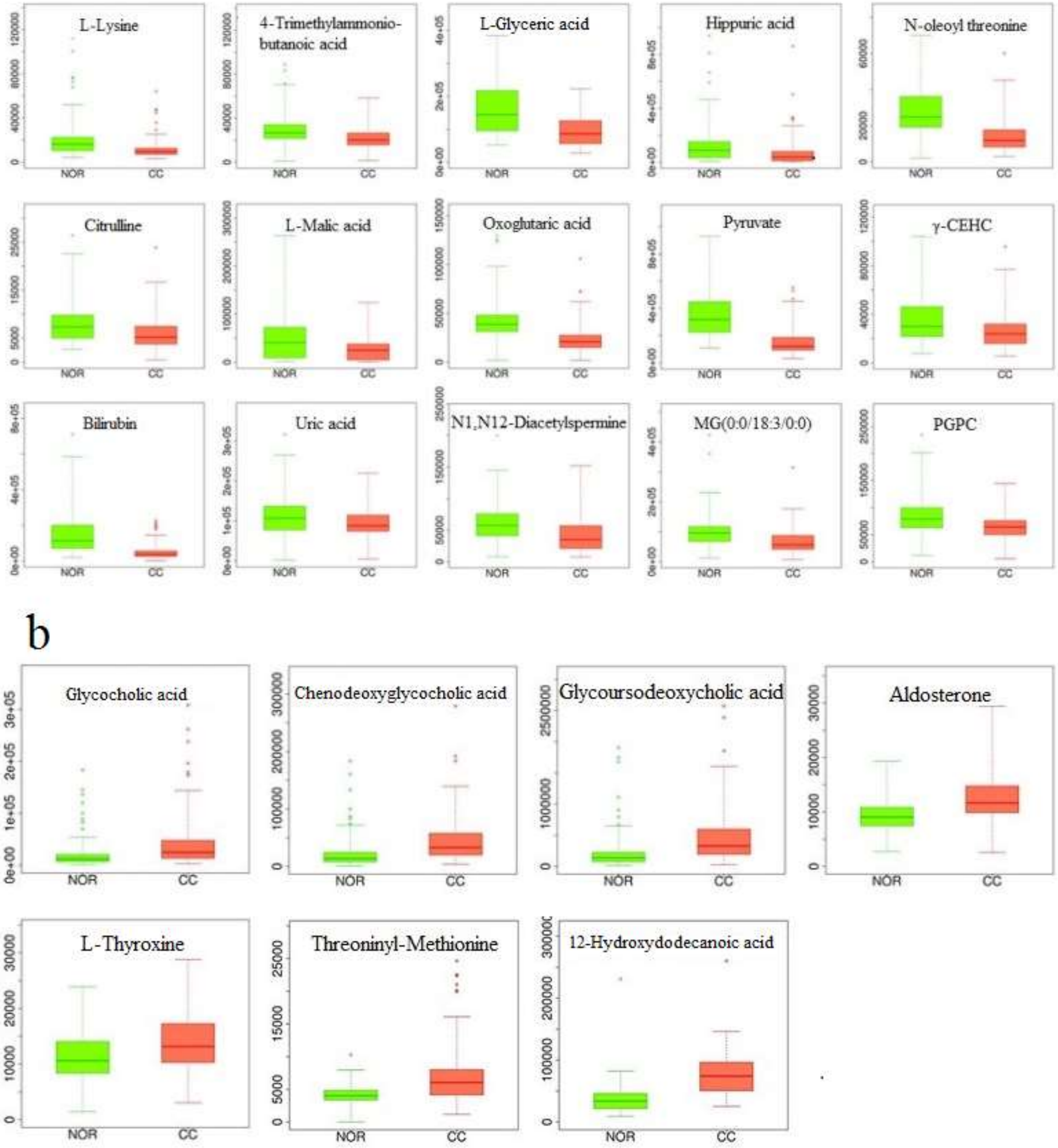


**Supplementary Figure S2:** (A) box plots for metabolites down-regulated in cervical cancer patients. (B).boxplot plots for metabolites up-regulated in cervical cancer patients.

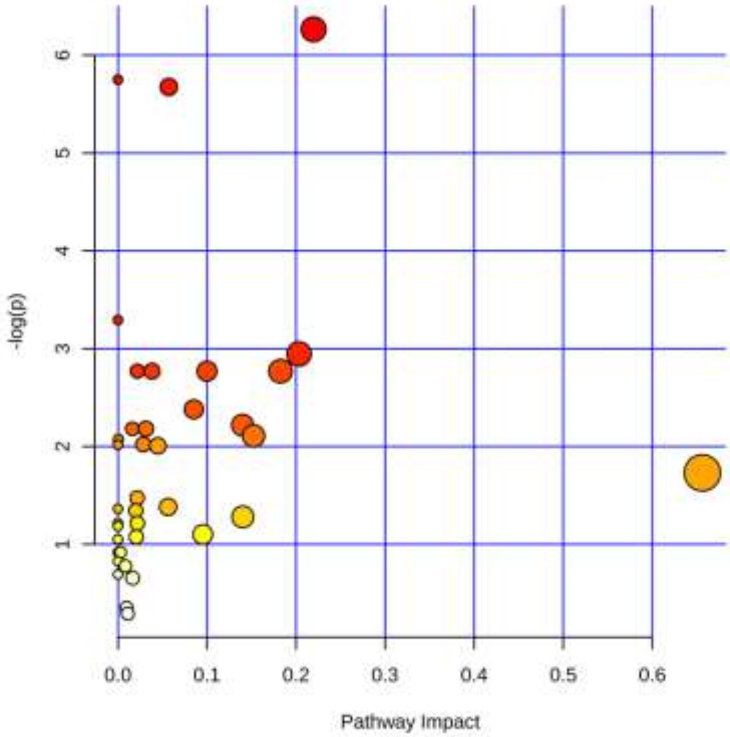

**Supplementary Figure S3:** Summary of the pathway analysis.
